# The impact of combined spinal-epidural analgesia (CSEA) on postpartum depression: A Mendelian randomization study

**DOI:** 10.1080/24740527.2026.2617362

**Published:** 2026-03-02

**Authors:** Mingyue Zhang, Shaoxing Liu

**Affiliations:** Department of Anesthesiology, Chengdu Second People’s Hospital, Chengdu, Sichuan, China

**Keywords:** combined spinal-epidural analgesia, postpartum depression, Mendelian randomization

## Abstract

**Background:**

Postpartum depression (PPD) develops within the first few weeks or months following delivery and causes severe emotional and psychological problems. Pain has been closely linked to the occurrence of depression. Observational studies have suggested that effective pain relief during childbirth can reduce the incidence of PPD. However, these studies are fraught with numerous confounding factor. Combined spinal-epidural analgesia (CSEA) is a commonly used pain relief method during childbirth. It is currently unclear whether a causal relationship exists between CSEA and PPD.

**Methods:**

An analysis was conducted using five methods in Mendelian randomization (MR) to study the use of CSEA during childbirth and PPD. The data were obtained from the United Kingdom Biobank database for CSEA and from FinnGen for PPD. The analysis included MR-Egger, weighted median, inverse variance weighted (IVW), simple mode, and weighted mode. We then conducted exposure heterogeneity testing using Cochran’s *Q* statistics and assessed the pleiotropy of exposure single nucleotide polymorphisms (SNPs) using MR-Egger.

**Results:**

IVW odds ratio (OR) = 0.978; 95% confidence interval (CI) 0.407, 1.031; *P* = 0.408. The results of the weighted median (OR = 1.035; 95% CI 0.995; 1.118, *P* = 0.377), simple mode (OR = 0.929; 95% CI 0.773, 1.116; *P* = 0.435), and weighted mode (OR = 0.995; 95% CI 0.930, 1.065; *P* = 0.888) suggest that there is no significant link between CSEA and PPD.

**Conclusion:**

We have concluded that there is no causal link between CSEA and PPD. This information can assist clinical professionals in gaining a better understanding of this condition.

## RÉSUMÉ

**Contexte**: La dépression post-partum survient au cours des premières semaines ou des premiers mois après l’accouchement et entraEEne de graves problèmes émotionnels et psychologiques. La douleur a été étroitement associée à la survenue de la dépression. Des études observationnelles indiquent qu’un soulagement efficace de la douleur pendant l’accouchement peut réduire l’incidence de la dépression post-partum. Cependant, ces études sont grevées de nombreux facteurs de confusion. L’analgésie spino-épidurale combinée est une méthode de soulagement de la douleur couramment utilisée pendant l’accouchement. On ignore encore s’il existe une relation causale entre l’analgésie spino-épidurale combinée et la dépression post-partum.

**Méthodes**: Une analyse a été menée à l’aide de cinq méthodes de randomisation mendélienne(MR) afin d’étudier l’utilisation de l’analgésie spino-épidurale combinée pendant l’accouchement et la dépression post-partum. Les données ont été obtenues à partir de la base de données UK Biobank pour l’analgésie spino-épidurale combinée et celles concernant la dépression post-partum provenaient de FinnGen. L’analyse comprenait les méthodes MR-Egger, médiane pondérée, pondération par l’inverse de la variance (IVW), mode simple et mode pondéré. Des tests d’hétérogénéité de l’exposition ont ensuite été réalisés à l’aide de la statistique Q de Cochran, et la pléiotropie des polymorphismes nucléotidiques simples associés à l’exposition a été évaluée par la méthode MR-Egger.

**Résultats**: Pour la méthode de pondération par l’inverse de la variance, le rapport de cotes était de 0,978 (IC à 95 % : 0,407 - 1,031; P = 0,408). Les résultats de la médiane pondérée (RC = 1,035; IC à 95 % : 0,995 - 1,118, P = 0,377), du mode simple (RC = 0,929; IC à 95 % : 0,773 - 1,116 ; P = 0,435) et du mode pondéré (RC = 0,995; IC à 95 % : 0,930 - 1,065; P = 0,888) suggèrent qu’il n’existe pas d’association significative entre l’analgésie spino-épidurale combinée et la dépression post-partum.

**Conclusions**: Il a été conclu qu’il n’y a pas de relation causale entre l’analgésie spino-épidurale combinée et la dépression post-partum. Cette information peut aider les cliniciens à mieux comprendre cette affection.

## Introduction

Approximately 140 million babies are born globally each year, making the safety and health of pregnant women during the perinatal period a significant public health concern.^1^ Postpartum depression (PPD) refers to severe depressive symptoms or a typical depressive episode occurring at any time during the first year after childbirth in women.^2^ The global incidence rate of PPD is between 15% and 25%.^3^ Typical PPD occurs within 6 weeks after childbirth and can resolve spontaneously within 3 to 6 months.^2^ However, severe cases can persist for 1 to 2 years, and there is a recurrence rate of 20% to 30% with subsequent pregnancies. Its clinical features include low mood, anxiety, fatigue, insomnia, changes in appetite, decreased interest in the baby, and maternal role insecurity, among others.^4,5^ Previous research has indicated that PPD can have significant effects on both the mother and the offspring, such as lower cognitive function in the offspring and a higher incidence of insecure attachment.^5^ Therefore, understanding the potential factors leading to PPD is crucial.

There are many potential factors contributing to the occurrence of PPD: psychosocial and environmental factors (such as socioeconomic status, social support, and marital relationship) and biological factors (such as hormones, inflammation, genetics, and pain) have been shown to be closely associated with the development of PPD.^6–8^ It is well established that pain is an independent risk factor for the onset and progression of depression.^9^ The childbirth process involves intense pain, which can have many adverse effects on both the mother and the fetus, including chronic pain, postpartum stress syndrome, and negative psychological and physiological consequences.^6–8^ Some studies have suggested that women who experience significant pain during pregnancy, such as back or pelvic pain, may be at a higher risk of developing PPD.^10–12^

### Effective pain management during labor is therefore of clinical importance

Combined spinal-epidural analgesia (CSEA) is a widely used and effective method for labor analgesia. It provides rapid and sustained pain relief by administering local anesthetics into the subarachnoid and epidural spaces.^13,14^ Its advantages include allowing the mother to remain conscious and avoiding systemic effects associated with general anesthesia.^15^

Given the established link between pain and depression, as well as the efficacy of CSEA in alleviating labor pain, researchers have sought to investigate whether effective labor analgesia might reduce the risk of PPD. However, existing evidence remains inconsistent. Some observational and longitudinal studies suggest that neuraxial labor analgesia is associated with a lower incidence of PPD,^16–19^ whereas other studies, including randomized controlled trials, have failed to confirm this protective effect.^20–22^ These discrepancies may stem from confounding factors inherent in observational designs, such as socioeconomic status, prenatal mental health, and personal birth preferences.

Mendelian randomization (MR) is a genetic epidemiological approach that uses genetic variants as instrumental variables (IVs) to infer causal relationships between exposures and outcomes.^23^ Because genetic variants are randomly assigned at conception and generally independent of postnatal confounding factors, MR can provide more robust evidence of causality compared to conventional observational studies. In this study, we utilized MR to examine whether there is a causal relationship between the use of CSEA during childbirth and the subsequent risk of developing PPD, using genetic data from large-scale biobanks. This approach aims to overcome the limitations of prior observational research and offer clearer insights into the potential role of labor analgesia in PPD etiology.

## Methods

### Overall study design

**Figure 1.**
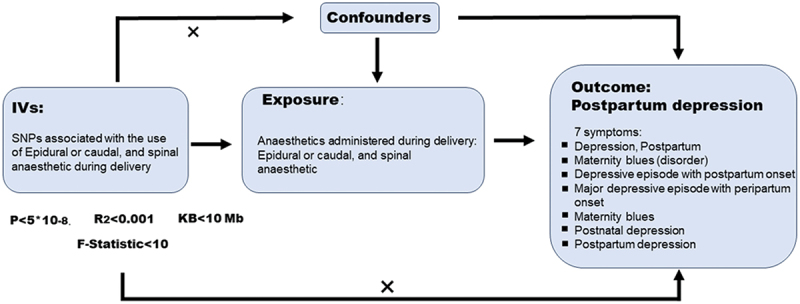
Overview diagram of MR analyses. Genetic data on CSEA and PPD.

MR is a data analysis technique used for assessing causal inference in epidemiology. It evaluates the causal relationship between the exposure of interest and the outcome of concern by employing genetic variations as IVs. Genetic loci are determined at the moment of conception and remain unaffected by postnatal factors. This determinism is the primary reason MR utilizes genetic loci as analytical tools. Selecting appropriate IVs requires satisfying three conditions: First, IVs must be independent of other confounding factors influencing the outcome variable. Second, IVs should have a close association with the exposure factor. Third, there should be no correlation between IVs and the outcome variable. To identify IVs meeting these criteria, we established criteria based on the values of *P, R*^2^, and *F* statistics. Since fewer single nucleotide polymorphisms (SNPs) were retained after applying the condition of *P* < 5 × 10^−8^, we modified the *P* value requirement to *P* < 5 × 10^−6^. Subsequently, SNP independence settings were implemented. We performed Clump on all SNPs with the conditions set at linkage disequilibrium (LD) *R*^2^ < 0.001 and kilobase (KB) < 10 Mb. Finally, SNP statistics strength settings were applied, requiring *F* statistics >10 for all SNPs. Due to the relatively limited number of genomewide significant SNPs (*P* < 5 × 10^−8^) associated with CSEA use, we employed a more lenient threshold of *P* < 5 × 10^−6^ to identify a sufficient set of IVs for robust MR analysis. All selected SNPs were subsequently clumped for independence, and their strength was confirmed using *F* statistics >10 to minimize the risk of weak instrument bias. The overall study design is illustrated in Figure 1.

We utilized data from the UK Biobank, which is currently one of the most renowned and open biobanks worldwide, specifically data related to “anesthetics administered during delivery” (Data Field 41,219). This data set collected information from 16,657 women who underwent childbirth between June 2013 and March 2023. Among them, we used the genomewide association study (GWAS) summary statistics data of 320 women who received CSEA as our exposure factor SNPs (Phenotype Code: 41,219_6). Additionally, we obtained PPD GWAS data (EFO_0007453) from the FinnGen database, which includes data from 226,707 participants, comprising 11,711 cases and 214,996 controls. This phenotype is defined based on *International Classification of Diseases*, revisions 10 and 9 (ICD-10/ICD-9) diagnosis codes for PPD, corresponding to a clinical diagnosis of depression in the postpartum period. The data sources are summarized in Table 1.

**Table 1.** Characteristics of data sources for Mendelian randomization analysis.

Trait	Source	Sample size	Cases/controls or *N*	Ancestry	Note
Exposure: CSEA	UK Biobank (Field 41,219)	16,657 women	320 (CSEA users)	Predominantly European	GWAS summary stats for phenotype code 41219_6
Outcome: PPD	FinnGen (Release R9)	226,707 individuals	11,711 cases/214,996 controls	Finnish	Phenotype code EFO_0007453

#### Genetic data harmonization and ancestry considerations

The exposure (CSEA) and outcome (PPD) GWAS summary statistics were derived from the UK Biobank and FinnGen databases, respectively. Both resources primarily comprise individuals of European genetic ancestry. Specifically, the UK Biobank cohort is predominantly of British descent, whereas FinnGen participants are of Finnish ancestry. Although both are European populations, differences in genetic substructure and LD patterns can exist between them, which, if unaddressed, could introduce bias in cross-population MR analyses.^20^ To minimize potential bias arising from population stratification and differing LD structures, we implemented the following strategies: Ancestry consistency: We restricted our analysis to genetic variants and summary statistics from these European-ancestry cohorts, avoiding trans-ancestry comparisons that carry higher risk of bias.

LD reference panel for clumping: The clumping procedure to ensure independence among IVs (SNPs) was performed using a European ancestry LD reference panel from the 1000 Genomes Project. This ensures that the LD estimates used to define SNP independence are appropriate for the population under study.

Robust harmonization: During the harmonization step between exposure and outcome data sets, we carefully aligned alleles based on effect allele frequency and removed palindromic SNPs with ambiguous strand orientation, as well as SNPs with incompatible alleles. This step is crucial for ensuring that effect estimates correspond to the same genetic effect across data sets.

Though these measures substantially mitigate concerns related to population differences, we acknowledge that subtle variations in genetic architecture between the British and Finnish subpopulations may persist as a minor limitation. However, the standard MR framework employed, coupled with the use of a consistent ancestral LD reference, is widely accepted for analyses within broad continental groups and is considered robust for causal inference in this context.^24,25^

#### Phenotype definitions in source GWAS

The exposure phenotype “anesthetics administered during delivery” (Data Field 41219) was derived from hospital inpatient records linked to the UK Biobank, which use ICD-10 and OPCS-4 coding systems. The specific code for “combined spinal-epidural [analgesia]” was identified within this field. The outcome phenotype for PPD (EFO_0007453) in the FinnGen consortium was defined based on nationwide registries, primarily using ICD-10 diagnosis codes (e.g., F53.0) from hospital discharge, outpatient, and primary care records.^26^

### Statistical analysis

We conducted a merge of the selected SNPs related to women who received CSEA during childbirth and SNPs associated with PPD (Supplementary Material 1). Within the PPD SNPs, we filtered out those related to CSEA. Subsequently, a harmonization process was carried out to align the allele directions of exposure SNPs and outcome SNPs. Based on effect allele frequency size, we excluded palindromic SNPs that could not determine direction and incompatible SNPs. In the end, we identified a total of 76 relevant SNPs (Supplementary Material 2). Following this, we proceeded with MR estimation. MR estimation involved five methods: MR-Egger, weighted median, inverse variance weighted (IVW), simple mode, and weighted mode. IVW was the primary analysis method. We then conducted exposure heterogeneity testing using Cochran’s *Q* statistics and assessed the pleiotropy of exposure SNPs using MR-Egger. If no heterogeneity was observed, we employed a fixed effects mo if heterogeneity was present, a random effects model was utilized. The effects of CSEA on PPD are presented as odds ratios (ORs) with corresponding 95% confidence intervals (95% CIs). For the entire MR analysis, we used R 4.3.1 software,^27^ along with the integrated TwoSampleMR package.

## Results

Through MR analysis, it was found that there is no causal relationship between the use of CSEA for pain relief during childbirth and PPD (Table 2).

**Table 2.** Summary of results for five methods in MR analysis.

Method	Used of SNPs	OR	95% CI	*P*
MR-Egger	76	0.986	(0.921, 1056)	0.693
Weighted median	76	1.035	(0.959, 1.118)	0.377
Inverse variance weighted	76	0.978	(0.407, 1.031)	0.408
Simple mode	76	0.929	(0.773, 1.116)	0.435
Weighted mode	76	0.995	(0.930, 1.065)	0.888

**Figure 2.**
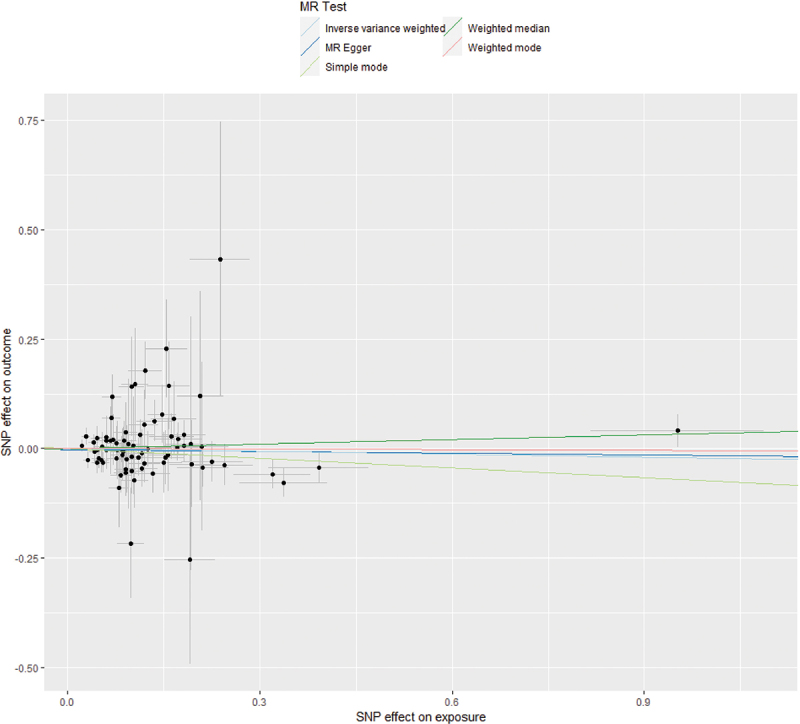
Scatter plots of MR analyses.

The IVW method is the primary approach for predicting the impact of CSEA on PPD. The results showed a *P* value of 0.408 for IVW, with an OR value of 0.978 and a 95% CI of 0.407 to 1.031. Therefore, this suggests that CSEA does not influence the occurrence or development of PPD. Similarly, the weighted median (OR = 1.035; 95% CI 0.995, 1.118; *P* = 0.377), simple mode (OR = 0.929; 95% CI 0.773, 1.116; *P* = 0.435), and weighted mode (OR = 0.995; 95% CI 0.930, 1.065; *P* = 0.888) all indicated that there was no statistically significant association between CSEA and PPD (Table 2, Figure 2).

### Heterogeneity analysis

**Figure 3.**
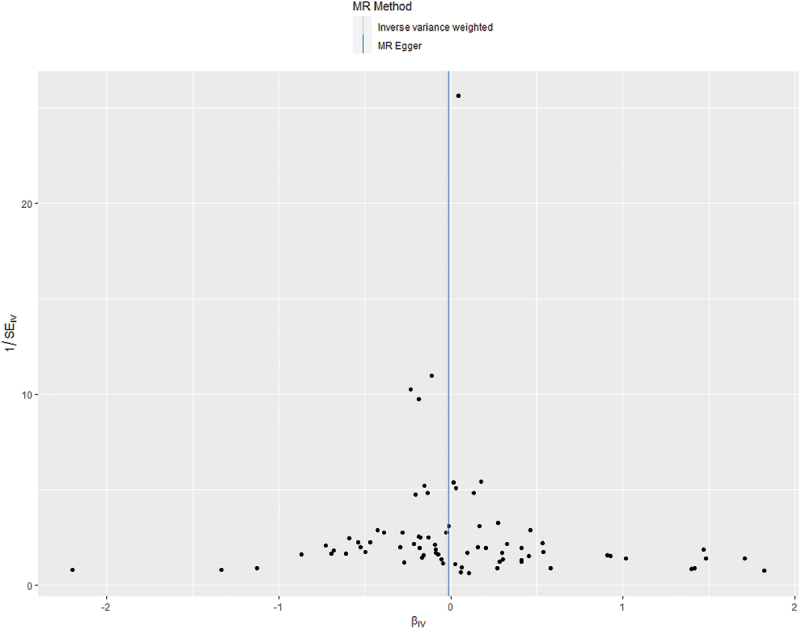
Funnel plot for examining heterogeneity of SNPs associated with CSEA.

We conducted an examination of horizontal pleiotropy related to SNPs associated with CSEA using the Cochran *Q* statistics method. The statistical analysis resulted in a *P* value of 0.454, indicating the absence of heterogeneity (Figure 3). The absence of significant heterogeneity suggests that the individual genetic instruments (SNPs) for CSEA provide consistent estimates of its effect on PPD, supporting the validity of using a fixed effects meta-analysis model for the IVW method.

### Horizontal pleiotropy analysis

We examined horizontal pleiotropy related to SNPs associated with CSEA using the MR-Egger method. The calculated *P* value was 0.691. Therefore, in the study aiming to predict the causal relationship between SNPs related to CSEA during childbirth and PPD, there was no influence from confounding factors (Figure 2). This suggests that the genetic instruments for CSEA are unlikely to influence PPD through pathways other than CSEA itself. Therefore, the core assumption of MR—that instruments affect the outcome only via the exposure—appears tenable in this analysis, lending credibility to the causal inference.

The consistent null findings across all five complementary MR methods, which make different assumptions about instrument validity (e.g., MR-Egger allows for some pleiotropy, and weighted median is robust to up to 50% invalid instruments), strengthens the inference that there is no detectable causal effect of CSEA on PPD, because the conclusion is not dependent on the assumptions of any single method.

## Discussion

In this study, we conducted an MR analysis to examine the causal relationship between the use of effective epidural analgesia during childbirth at the genetic level and the occurrence and development of PPD. The conclusion drawn from our analysis is that there is no statistically significant correlation between them. This finding differs from the results of many previous observational studies. By studying the impact of CSEA on PPD at the genetic level, we eliminated interference from various other confounding factors, providing a more direct indication of the causal relationship between CSEA and PPD.

Previous studies have indicated that there are various potential causes for the occurrence of PPD. These include rapid hormonal changes following childbirth, genetic factors, and social factors such as economic factors, and marital/family relationships.^9,28,29^

Chronic and acute pain are well-established independent risk factors for the development of depression.^9^ Childbirth is associated with severe pain, which has spurred investigation into whether effective intrapartum analgesia can mitigate the risk of subsequent PPD. However, the existing body of evidence has yielded inconsistent conclusions.

A meta-analysis by Mo et al., which included 19 observational studies involving 8758 parturients, found that perinatal pain increased the risk of PPD (OR = 1.43; 95% CI 1.23, 1.67), but the use of epidural analgesia during childbirth was associated with a significantly reduced risk (OR = 0.42; 95% CI 0.33, 0.55).^16^ Supporting this, a prospective cohort study reported a significantly lower incidence of PPD among parturients receiving epidural analgesia compared to those who did not.^21^ In contrast, a randomized controlled trial involving 130 parturients found no statistically significant difference in PPD rates at 6 weeks postpartum between the analgesia and nonanalgesia groups (27.7% vs. 16.9%).^22^ Similarly, a meta-analysis by Wang et al. concluded that neuraxial labor analgesia did not reduce the risk of PPD (OR = 0.84; 95% CI 0.58, 1.23).^30^ Exploring potential physiological mechanisms, Riazanova et al. assessed perinatal cortisol levels and found that though epidural analgesia reduced early postpartum pain and cortisol, its impact on PPD risk at 6 weeks was minimal.^31^

Due to ethical concerns, there have been few randomized controlled experiments exploring the relationship between labor analgesia and PPD to date. Observational research methods have been the primary approach in studying the connection between pain and PPD, but their reliability is questionable due to the presence of confounding variables, high heterogeneity, and limitations such as misclassification errors in the analysis process. The discrepancy between our null genetic findings and some positive observational associations may be explained by key methodological differences. MR estimates the lifelong effect of a genetic predisposition toward receiving CSEA, which is largely independent of the acute clinical and psychosocial context of childbirth. In contrast, observational studies measure the effect of the actual clinical administration of CSEA. This clinical exposure is subject to confounding by indication; for instance, women with higher pain sensitivity, preexisting anxiety, or specific socioeconomic profiles may be both more likely to opt for labor analgesia and have a different baseline risk of PPD.^21,22^ Furthermore, the positive experience of effective pain relief or negative perceptions of medical intervention could have immediate psychological impacts not captured by a genetic proxy. Therefore, our MR result suggests that the biological act of neuraxial blockade itself, as proxied by genetics, may not directly cause or prevent PPD. The associations seen in some observational studies are likely mediated or confounded by these nonbiological, situational, and psychological factors surrounding the decision to use and the experience of receiving analgesia.

Certainly, this study has its own limitations. First, a primary limitation of this study is the limited statistical power arising from the sample size of the exposure GWAS. The genetic instrument for CSEA was based on only 320 cases from the UK Biobank. This small sample size reduces the power to detect a causal effect of modest magnitude and results in wide confidence intervals for our estimates, as evidenced in our results. Therefore, our null finding should be interpreted with caution; it may represent a true absence of effect or a lack of power to detect a small but clinically relevant effect. Future studies with larger, dedicated GWAS for labor analgesia phenotypes are needed to confirm these findings. Second, the GWAS data used in this study from parturients who received CSEA cover a wide range, and many details about the anesthesia, such as the type and amount of local anesthetics used during CSEA, are not reflected in the GWAS. Third, because the genetic data in this study were obtained from biobanks comprising individuals of European ancestry (UK Biobank and FinnGen), the generalizability of our findings to parturients of other ethnicities and geographic regions may be limited. Genetic variants, their associations with CSEA use, and the genetic architecture of PPD may differ across populations. For example, previous studies have reported that Japanese parturients who received analgesia during vaginal delivery had a higher probability of developing PPD 6 months postpartum than those who did not receive analgesia during delivery (OR = 1.233; 95% CI 1.079, 1.409).^32^ The authors explained that this might be due to a maternal myth in Japanese culture, which states that labor pains during childbirth helps to form strong maternal instincts. This belief leads to fewer Japanese parturients using analgesia during childbirth; as a result, those who do use it may experience social and familial psychological pressures later on, making them more susceptible to PPD.^32^ Last, a fundamental characteristic and limitation of the MR design is that it estimates the biological effect of an exposure, isolated from the psychosocial context. Our analysis of genetically proxied CSEA cannot account for how cultural attitudes, social support, personal birth expectations, or the subjective experience of pain relief—all of which are potent modifiers of postpartum mental health—interact with the receipt of analgesia. For example, the cited study from Japan^3^5 highlights that the social meaning of labor analgesia can influence PPD risk. MR is not designed to capture these complex gene–environment or treatment context interactions. Therefore, our finding of no direct biological causal effect does not preclude the possibility that the clinical and experiential context of receiving CSEA significantly influences PPD risk in specific settings or populations. The study conducted an MR analysis to explore the relationship between CSEA and PPD. This research has shed light on the potential causes of PPD, which can aid clinical anesthesiologists, obstetricians, and psychiatrists in better understanding the condition. Additionally, this study opens the door for further investigation into highly correlated factors that contribute to PPD, ultimately helping to prevent it.

## Conclusion

In summary, using genetic data from European ancestry biobanks, our study does not provide evidence for a causal biological effect of CSEA on PPD. These findings highlight the need to consider nonbiological and contextual factors when interpreting observational links between labor analgesia and postpartum mental health. Future research with larger, diverse genetic data sets is warranted to confirm the generalizability of these results and to explore the complex interplay between medical intervention, psychology, and culture in the etiology of PPD.

## Data Availability

Data related to anesthetics administered during delivery (Data Field 41,219) from UK Biobank. Data related to postpartum depression (EFO_0007453) from the FinnGen database.
